# Synaptic biomarkers in CSF aid in diagnosis, correlate with cognition and predict progression in MCI and Alzheimer's disease

**DOI:** 10.1016/j.trci.2019.11.002

**Published:** 2019-12-09

**Authors:** Douglas Galasko, Meifang Xiao, Desheng Xu, Denis Smirnov, David P. Salmon, Nele Dewit, Jeroen Vanbrabant, Dirk Jacobs, Hugo Vanderstichele, Eugeen Vanmechelen, Alzheimer's Disease Neuroimaging Initiative (ADNI), Paul Worley

**Affiliations:** a Department of Neurosciences, University of California, San Diego, La Jolla, CA, USA; b Department of Neuroscience, Johns Hopkins University School of Medicine, Baltimore, MD, USA; c ADx NeuroSciences, Ghent, Belgium

**Keywords:** Alzheimer's disease, Cerebrospinal fluid, Biomarker, Synapse, Prognosis

## Abstract

**Introduction:**

Amyloid, Tau, and neurodegeneration biomarkers can stage Alzheimer's Disease (AD). Synaptic biomarkers may help track cognition.

**Methods:**

In cognitively normal controls, Mild Cognitive Impairment (MCI) and AD, we investigated CSF biomarkers in relation to cognitive measures and as predictors of cognitive and global decline.

**Results:**

There were 90 normal controls (mean age 73.0, 58% women), 57 MCI (mean age 74.3, 35% women), and 46 AD (mean age 70.7, 41% women). CSF Aβ1-42 and Neuronal Pentraxin 2 (NPTX2) were decreased, and CSF Tau, neurogranin, and SNAP25 increased in AD versus controls. Aβ1-42/Tau or NPTX2/Tau discriminated AD and controls best. NPTX2/Tau correlated strongly with cognition in AD and MCI and predicted a 2–3-year decline. We replicated findings in the ADNI cohort.

**Discussion:**

CSF synaptic biomarkers, particularly NPTX2, which regulates synaptic homeostasis, relate to cognition and predict progression in AD beyond Aβ1-42 and Tau. This is relevant for prognosis and clinical trials.

## Highlights

• CSF levels of synaptic biomarkers and their ratios to CSF Tau improved AD diagnosis
• CSF synaptic biomarkers, particularly NPTX2, predicted cognitive decline.
• Combining CSF biomarkers of neurodegeneration may improve diagnosis or prognosis.
• NPTX2 relates to interneuron-dependent circuit homeostasis, likely impaired in AD.

## 1 Background

A recently proposed research framework emphasizes biomarkers for amyloid, tau, and neurodegeneration (A,T,N) for diagnosis and staging of Alzheimer's Disease (AD) [1,2]. Neurodegeneration biomarkers investigated in AD include brain atrophy measured by MRI [3], decreased regional glucose metabolism assessed using fluorodeoxyglucose (FDG) PET [4], and increases in CSF proteins that may reflect damage, for example, Tau and neurofilament light (Nfl) [5,6]. These markers show changes in people with Mild Cognitive Impairment (MCI) and dementia due to AD compared to older cognitively normal subjects; however, they correlate relatively weakly with each other and show varying relationships to cognitive test scores and in classifying symptomatic AD [7].

Synaptic damage or dysfunction is a key pathological feature of AD that correlates with cognitive function in clinical-pathological studies and may link Tau and amyloid pathogenetic mechanisms [8]. Presynaptic proteins (e.g., SNAP25 [Synaptosomal nerve-associated protein 25]) [9] and dendritic proteins (e.g., neurogranin) [10,11] are increased in CSF in MCI and AD. A recent study showed AD-related increases in a panel of synaptic proteins measured by mass spectrometry in CSF [12]. Several proteomic analyses of CSF aimed at discovering AD biomarkers have identified changes in neuronal pentraxins, which play important roles in synaptic regulation [[13], [14], [15], [16]]. We reported that Neuronal Pentraxin 2 (NPTX2), a secreted synaptic protein that mediates homeostatic adaptation to increased excitability by enhancing inhibitory synaptic circuits [17,18], is markedly decreased in postmortem brain lysates and in CSF in MCI and AD [19].

The present study was designed to explore the cross-sectional relationship between synaptic biomarkers in CSF and standardized neuropsychological tests across normal cognition, MCI, and AD, and to determine the prognostic value of synaptic and other CSF biomarkers to predict cognitive decline.

## 2 Methods

### 2.1 Standard protocol approvals, registrations, and patient consents

Research protocols were reviewed and approved by the human subjects review board at the University of California, San Diego. Informed consent to participate in the study was obtained at enrollment into the Alzheimer's Disease Research Center (ADRC) longitudinal study from all participants or where appropriate their caregivers, consistent with California State law. Informed consent and IRB approval for ADNI participants are described at www.adni-info.org.

### 2.2 Subjects

#### 2.2.1 UCSD cohort

Subjects with NC, MCI, and mild AD followed in a longitudinal observational study at the UCSD Shirley-Marcos ADRC were studied based on the availability of CSF samples and longitudinal neuropsychological data. All subjects received comprehensive research assessments, including history from subject and informant, medical and neurological examination, Mini-Mental State Exam (MMSE), Clinical Dementia Rating (CDR), a standard neuropsychological test battery, laboratory blood tests, and brain MRI. Data were reviewed at enrollment and annually, and a research diagnosis was made by consensus conference among three Neurologists with extensive expertise in dementia. Consensus diagnoses were blind to CSF biomarker data. Diagnostic criteria for MCI and AD followed guidelines of the National Institute on Aging-Alzheimer Association (NIA-AA) working groups in 2011 but did not require an amyloid biomarker [20,21]. Neuropsychological test scores were used to classify MCI into amnestic or nonamnestic subtypes. Clinical and neuropsychological assessments were repeated annually, and follow-up consensus diagnoses were assigned.

Blood was drawn for DNA analysis, including apolipoprotein E (*APOE*) genotype, and subjects received research lumbar punctures with standardization of procedures, preanalytical preparation, and storage of CSF and plasma as previously described [19], and in accordance with recommended best practices [22]. In brief, CSF (15–25 mL) was collected by routine lumbar puncture early in the morning after overnight fasting. Samples were processed, aliquoted into 500 μL fractions in polypropylene microtubes, snap frozen, and stored at −80°C until assayed.

#### 2.2.2 ADNI

Data used in the preparation of this article were also obtained from the Alzheimer's Disease Neuroimaging Initiative (ADNI) database (adni.loni.usc.edu). The ADNI was launched in 2003 as a public-private partnership. The primary goal of ADNI has been to test whether serial magnetic resonance imaging (MRI), positron emission tomography (PET), other biological markers, and clinical and neuropsychological assessment can be combined to measure the progression of mild cognitive impairment (MCI) and early Alzheimer's disease (AD). Procedures for subject recruitment and biosample processing follow standardized operating procedures (SOPs). For up-to-date information, see www.adni-info.org.

### 2.3 Cognitive tests

#### 2.3.1 UCSD cohort

We analyzed global cognitive function with the Mattis Dementia Rating Scale (MDRS) [23], and learning and memory with the California Verbal Learning Test (CVLT) [24] or its updated version (CVLT II) [25]. These tests detect changes in the earliest stages of AD [26] and remain sensitive to the disease throughout its course. The MDRS assesses attention, initiation, and perseveration of behavior, conceptualization, constructional praxis, and memory, and scores range from 0–144. The CVLT is a rigorous test of verbal learning and memory that requires learning a 16-item list of words over five presentation-recall trials, recall of the words after short or long delay intervals, and then recognizing the words as members of the list. We examined measures of total recall over trials 1–5 and the sum of short- and long-delay recall conditions. These measures were pooled across the CVLT and CVLT II, as they are highly correlated across both versions [26]. As a global outcome measure, we examined the CDR sum of boxes (CDR-sb) [23], which combines ratings obtained from patients and an informants in six categories of function (‘boxes’), each of which is scored from 0 (best), through 0.5, 1, 2 or 3 (worst).

#### 2.3.2 ADNI

The Rey Auditory Verbal Learning Test (RAVLT) [25] (word list recall) Alzheimer's Disease Assessment Scale-cognitive (ADAS-cog) [27] (global cognitive test) and CDR-sb were used, providing close comparability with measures in the UCSD cohort.

### 2.4 CSF biomarkers

#### 2.4.1 UCSD subjects

Assays were run by experienced laboratory technicians, blind to clinical diagnostic information. Levels of Aβ1-42, Tau, and neurogranin in CSF were analyzed in the facilities of ADx (Gent, Belgium) using ELISA assays developed by ADx and commercialized by EUROIMMUN AG (Lübeck, Germany) [28,29]. NPTX2 was measured using a research-grade sandwich ELISA that we have described and validated [15]. CSF samples were run in duplicate in randomized order (specified by DG and HV) on each ELISA plate. Each plate contained a high and low reference standard derived from pools of CSF in addition to calibration curves using peptide or protein standards. *APOE* genotyping was performed using PCR methods, as previously described [26].

The SNAP25 is an ADx home-brew Single Molecule Analysis (Simoa) bead-based immunoassay. In brief, an N-terminal acetylated specific monoclonal (ADx404) was used to capture SNAP25 from CSF, and a biotinylated detector antibody with an epitope from L26-L33 (ADx405) was used as a capture antibody. A synthetic peptide corresponding to amino acids A(Ac)2-K40 was used as a calibrator, covering the range of 2.5–100 pg/mL. The assay was run using a fully-automated protocol on a Simoa HD-1 Analyzer (Quanterix, Cambridge, MA). Assay details are described in Supplementary data.

#### 2.4.2 ADNI

CSF Aβ1-42 and Tau were measured by Elecsys assays [30]. CSF NPTX2 was measured as part of a proteomic analysis using Multiple Reaction Monitoring (MRM) [14], and we used normalized data for the peptide NPTX2_TESTLNALLQR. We selected ADNI participants with available data for these CSF biomarker analytes at baseline. We omitted neurogranin and SNAP25 because only about 50% of overlapping subjects had these data. CSF Neurofilament light (Nfl), measured by ELISA (Uman Diagnostics, Inc), was available for 97% of subjects. We included Nfl in analyses since it has been shown to predict cognitive decline in ADNI and other studies [31,32].

### 2.5 Data analysis

For the UCSD cohort, demographic variables, *APOE ϵ*4 frequencies, cognitive test scores, and CSF biomarkers were compared across AD, MCI, and CN groups. Continuous variables were analyzed with one-way ANOVA, and if significant, followed by pairwise comparisons using Tukey's HSD posthoc tests. Categorical variables were analyzed with a 3-group Fisher Exact Test, and if significant, followed by posthoc pairwise comparisons with Fisher Exact tests. Sensitivity and specificity to distinguish AD and control subjects were calculated for each CSF biomarker using Receiver Operating Characteristic (ROC) curve analysis. 95% confidence intervals for the area under the curve (AUC) were computed with 2000 stratified bootstrap replicates. DeLong's test for two correlated ROC curves [33] was used to compare AUCs of single biomarkers with their ratios. Correlations between different CSF biomarkers were examined in all subjects and each subgroup (control, MCI, and AD) using Pearson or Spearman analysis, as appropriate. Linear regression was used to examine the relationship between the biomarkers and cognitive performance on the MDRS and CVLT. Separate models for each biomarker, with covariate terms for age, sex, education, and *APOE ϵ*4 status, were used to examine the unique contribution of the biomarker to predictions of cognition. These relationships were also examined in people classified as amyloid positive or negative based on CSF Aβ1-42, using cutoffs specific to the UCSD and ADNI cohorts [30]. Additional models included terms for each biomarker and all the covariates together to compare the unique contribution of each biomarker in relation to others. Linear mixed models were used to examine which biomarkers predicted cognitive decline over up to 3 years of followup. Analyses were carried out using R version 3.5.2.

Similar methods were used for the ADNI data to compare baseline clinical and cognitive tests and biomarkers in controls, MCI, and AD, and to examine predictors of cognitive decline for up to 4 years of followup in MCI and AD. For ADNI, CSF biomarker data were log transformed as needed.

## 3 Results

### 3.1 UCSD cohort

Demographic, genetic, cognitive, and CSF biomarker data are shown in Table 1. The NC (n = 90), MCI (n = 57), and AD (n = 46) groups were similar in age, although AD patients, on average, were slightly younger than MCI and NC. Groups did not differ significantly in education. The NC group had a higher percentage of women than the two patient groups. As expected, MMSE, MDRS, and CVLT scores were worse (lower) for AD than MCI, and for MCI than NC. The *APOE ϵ*4 frequency was higher in AD than NC. Levels of CSF Aβ1-42 were lower in AD than in MCI or NC, and lower in MCI than in NC (Table 1). Tau in CSF was higher in AD than in MCI or NC, while MCI and NC did not differ. SNAP25 and neurogranin showed trends for increased levels in AD and MCI relative to NC, but group differences were not significant. CSF NPTX2 was decreased in MCI and AD relative to NC.

**Table 1:** Demographic, cognitive and biomarker data: UCSD cohort

	AD (n = 46)	MCI (n = 57)	NC (n = 90)	*P* value
Age (years)	70.7 ± 9.4	74.3 ± 6.5	73.0 ± 5.2	.025∗
Female, N (%)	19 (41)	20 (35)	52 (58)	.018†
Education (years)	15.5 ± 3.6	16 ± 2.9	16.7 ± 2.4	.092
MMSE (0–30)	23.5 ± 4	27.9 ± 2	29.3 ± 1	<.001∗^,^†^,^‡
MDRS (0–144)	114.3 ± 18.3	134.6 ± 5.3	139.6 ± 3.4	<.001∗^,^†^,^‡
CVLT trials 1–5 (0–80)	19.6 ± 6.6	34.0 ± 9.3	45.8 ± 10.3	<.001∗^,^†^,^‡
CVLT Delayed Recall (0–16)	2.6 ± 2.8	9.7 ± 6.3	19.3 ± 6.2	<.001∗^,^†^,^‡
CDR Sum of Boxes (0–18)	5.8 ± 2.7	1.6 ± 1.5	0.2 ± 0.6	<.001∗^,^†^,^‡
*APOE ϵ*4 % positive	30 (67)	29 (53)	35 (40)	.012‡
Aβ1-42 (pg/mL)	369 ± 146.5	530.9 ± 287.4	690.3 ± 291.4	<.001∗^,^†^,^‡
Tau (pg/mL)	774.9 ± 695.9	508 ± 298.8	380.2 ± 211.1	<.001∗^,^‡
Aβ1-42/Tau	0.7 ± 0.6	1.5 ± 1.3	2.4 ± 1.5	<.001∗^,^†^,^‡
NPTX2 (pg/mL)	715.1 ± 426.6	826.5 ± 474.4	1075 ± 504.8	<.001†^,^‡
SNAP 25 (pg/mL)	36.0 ± 15.6	34.9 ± 15.5	32.1 ± 9.8	.223
Neurogranin (pg/mL)	347.6 ± 235.6	332.2 ± 199.9	324.5 ± 163.4	.809

NOTE. The cohort was 95% White (of which 4% were Hispanic), 3% Asian, <1% each Black, American Indian, Other. Results are presented as mean ± standard deviation.

Abbreviations: AD, Alzheimer's Disease, MCI, Mid Cognitive Impairment, NC, normal cognition; MMSE, Mini-Mental State Examination; MDRS, Dementia Rating Scale; CVLT, California Verbal Learning Test; CDR, Clinical Dementia Rating; NPTX2, Neuronal Pentraxin 2; SNAP25, Synaptosomal-associated protein 25.

∗ Posthoc difference (*P* < .05) between MCI and AD.

† Posthoc difference (*P* < .05) between NC and MCI.

‡ Posthoc difference (*P* < .05) between NC and AD.

**Fig. 1:**
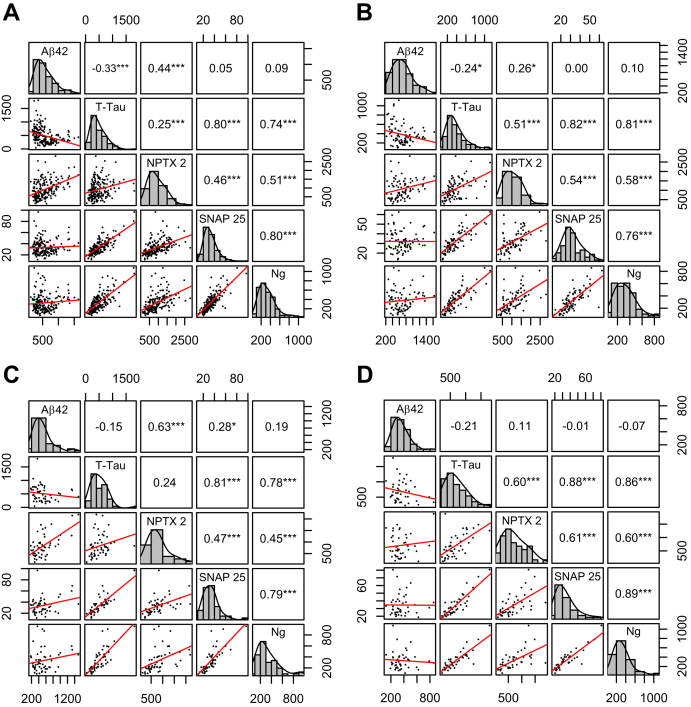
Relationship of biomarkers across diagnoses. Correlations between biomarker in the overall sample (A), or restricted to Normal Cognition (B), MCI (C), or AD patients (D). On the diagonal are labeled histograms for each biomarker. On the lower-left half of the plot, each scatterplot corresponds to the biomarker vertically above it on the x-axis and the biomarker horizontally to the right on the y-axis. The upper-right half of the plot shows the *R* correlation coefficients for each biomarker pair, with stars denoting the level of significance. *P < .05, **P < .01, ***P < .001.

Correlations among the CSF biomarkers are shown in Fig. 1 across all subjects and separated by group (NC, MCI, AD). NPTX2 correlated with Aβ1-42 overall and specifically in the MCI group, while other synaptic biomarkers (SNAP25 and neurogranin) did not. In contrast, NPTX2 showed a moderate relationship with Tau, SNAP25, and neurogranin, whereas SNAP 25, neurogranin, and Tau were strongly correlated (Rs from 0.7–0.8).

ROC analyses comparing NC and AD subjects showed that individual biomarkers varied in their overall classification accuracy (selected ROC curves are shown in Supplementary Fig. 1 and data in Supplementary Table 1). Aβ1-42 and Tau yielded the highest AUC levels of individual biomarkers. We also examined the classification accuracy of ratios of Aβ1-42/Tau, and synaptic biomarkers/Aβ1-42 or Tau. The best classification accuracy between AD and NC was for the NPTX2/Tau ratio (AUC [95% CI] = 0.937 [0.888–0.986]), although additional combinations also yielded high AUCs. We compared AUCs for individual synaptic biomarkers to AUCs for their ratios and found that NPTX2/Tau improved classification compared to NPTX2 alone (*P* = 4.1e-7) or Tau alone (*P* = .003). SNAP25/Aβ1-42 (*P* = 2.6e-10) or SNAP25/Tau (*P* = 2.5e-9) improved classification compared to SNAP25 alone. Similarly, neurogranin/Aβ1-42 (*P* = .007) and neurogranin/Tau (*P* = 7.1e-16) improved classification compared to neurogranin alone. Both SNAP25/Tau (*P* = .04) and neurogranin/Tau (*P* = .005) improved classification compared to Tau alone. Using the optimal ROC-derived threshold for Aβ1-42, 30 of the 57 patients with MCI (53%) were below the threshold, indicating presumed amyloid positivity. When the Aβ1-42/Tau ratio was used, 28 of 54 (52%) of the MCI patients were AD-like (high ratio), and when the NPTX2/Tau ratio was used, 29 of 54 (54%) of the MCI patients were classified as AD-like (low ratio). Further ROC analyses comparing the CSF biomarkers in NC and stable or progressing MCI showed similar findings to NC versus AD (Supplementary Fig. 2).

**Fig. 2:**
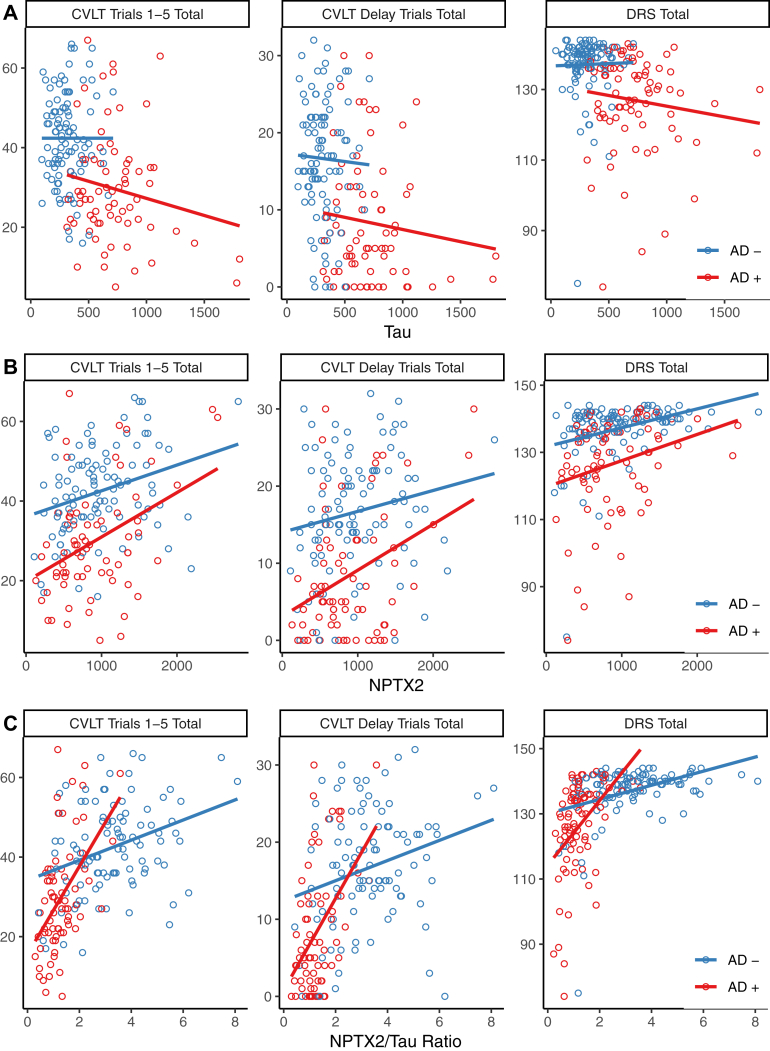

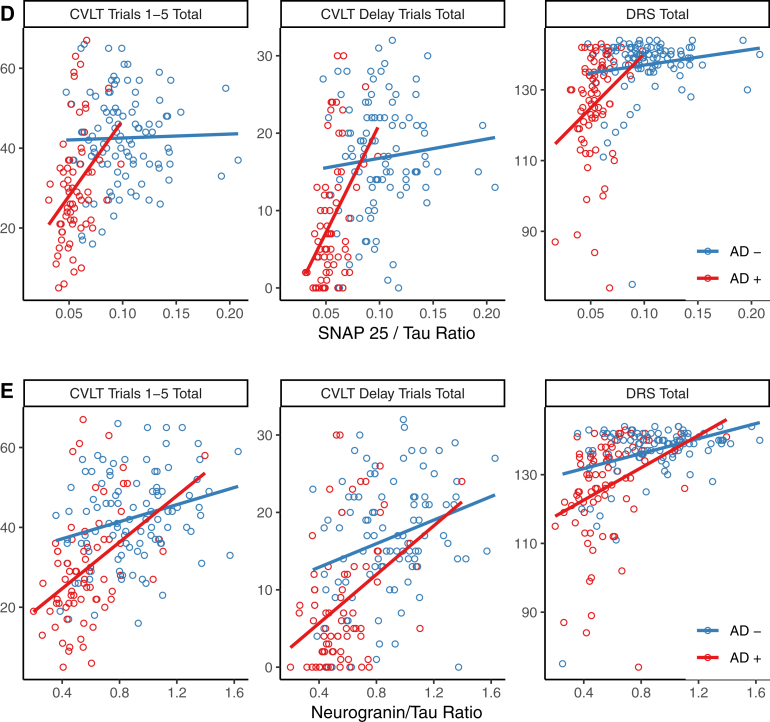
Biomarker prediction of cognitive measures. Correlations of Tau (A), NPTX2 (B), NPTX2/Tau ratio (C), SNAP24/Tau ratio (D), and neurogranin/Tau ratio (E) with cognition assessed with the California Verbal Learning Test (CVLT) immediate recall sum of trials 1–5, the CVLT sum of short and long delay free recall, and the Dementia Rating Scale (MDRS). Participants were dichotomized into “AD-like” (red) or not (blue) based on their CSF Aβ1-42/Tau ratio using the ROC derived cutoff. Linear regression lines for the two groups are plotted in the same colors. The models and effects of biomarkers and other predictors are fully presented in Supplementary Table 2.

Correlations between various CSF biomarkers and memory (CVLT Immediate and Delayed recall) and global cognition (MDRS) were examined across all subjects and after dividing subjects into those likely to have underlying AD or not based on Aβ1-42/Tau ratios (Fig. 2). Models assessing the added contribution of each synaptic biomarker individually after controlling for age, sex, education, *APOEϵ*4, Aβ1-42 levels, and Tau levels are shown in Supplementary Table 2a-c. NPTX2 and neurogranin both strongly contributed to the prediction of all cognitive measures in the entire sample, as well as in the subset with AD defined by Aβ1-42/Tau. Both NPTX2 and neurogranin predicted cognition in nonAD subjects defined by Aβ1-42/Tau on the CVLT immediate recall and MDRS, though only neurogranin significantly predicted CVLT delayed recall. SNAP25 predicted CVLT delayed recall in the whole sample and in AD (low Aβ1-42/Tau), but not in nonAD-like participants.

**Fig. 3:**
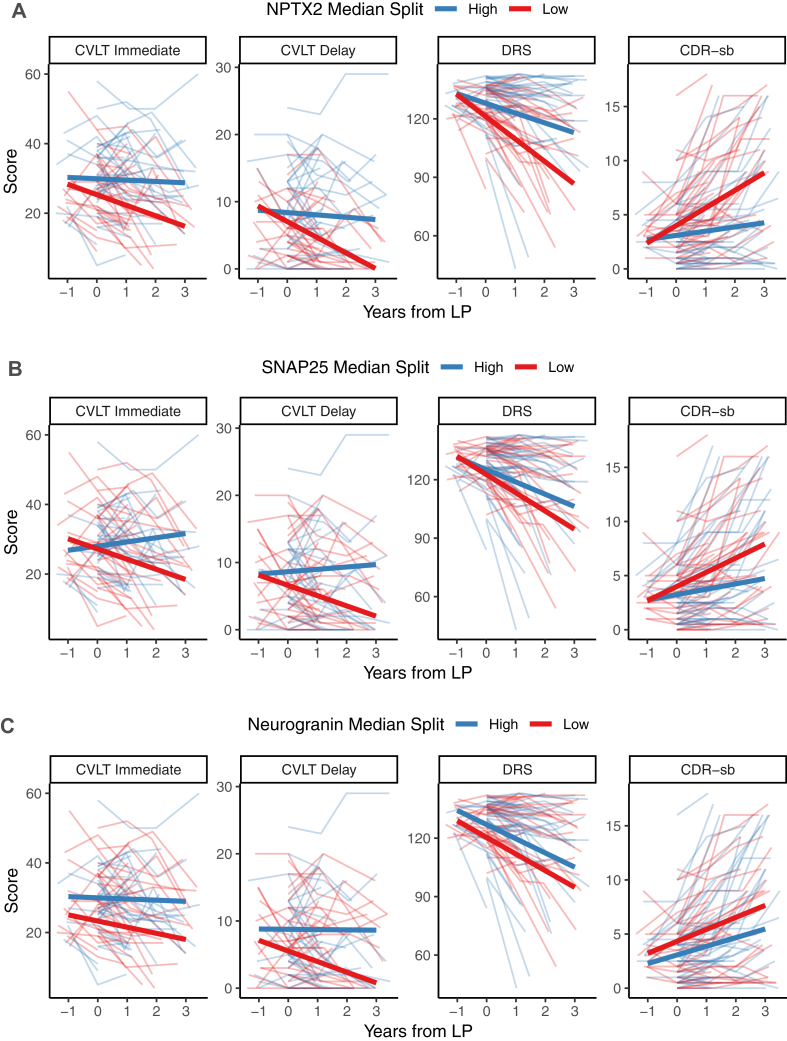
Biomarker prediction of longitudinal progression. Progression on the CVLT (immediate and delay), MDRS, and CDR Sum of Boxes (CDR-sb) divided via a median split of each CSF biomarker, NPTX2 (A), SNAP25 (B), and neurogranin (C), in subjects with diagnoses of MCI or AD. Raw data of individual participants is shown in the background, overlaid with predictions from longitudinal mixed-effect models, adjusted for demographics, Aβ1-42, and Tau. Note that in the model, each biomarker was treated as a continuous variable, but was dichotomized by median split for these graphical purposes only. The models and effects of biomarkers and other predictors are fully presented in Table 2.

The predictive value of synaptic or neurodegeneration CSF biomarkers for longitudinal change during up to four years of follow-up on the MDRS, CVLT (immediate and delayed recall) and CDR-sb in patients with MCI or AD was examined using linear mixed effect models that included demographics, *APOE ϵ*4 and CSF Aβ1-42 and Tau levels (selected relationships are shown in Fig. 3, and all relationships are shown in Table 2) and then added each synaptic marker to this model. NPTX2 levels had added significant predictive value for the rate of decline of all four cognitive measures, while SNAP25 only predicted decline on CVLT Immediate Recall and neurogranin only predicted decline on CVLT Delayed Recall.

**Table 2:** Models predicting longitudinal change in the UCSD cohort

Cognitive or clinical measure of change								
	CVLT trials 1–5		CVLT delay total		MDRS total		CDR-sb	
	Beta ± Std. Error	*P* value	Beta ± Std. Error	*P* value	Beta ± Std. Error	*P* value	Beta ± Std. Error	*P* value
Age	−0.104 ± 0.075	.174	0.019 ± 0.041	.641	0.442 ± 0.125	.001	−0.025 ± 0.021	.24
Sex	−1.109 ± 1.168	.350	0.013 ± 0.637	.984	0.571 ± 1.987	.776	0.369 ± 0.312	.244
Education	0.092 ± 0.200	.649	0.04 ± 0.109	.716	0.110 ± 0.283	.699	0.016 ± 0.045	.726
APOE e4 APOE	–1.274 ± 1.221	.304	0.126 ± 0.671	.852	1.54 ± 2.075	.463	0.198 ± 0.334	.556
Aβ1-42	1.711 ± 0.740	.025	1.531 ± 0.393	<.001	2.164 ± 1.138	.064	–0.174 ± 0.194	.375
Tau	1.122 ± 0.722	.128	0.03 ± 0.396	.94	–3.055 ± 1.104	.007	0.481 ± 0.175	.008
Above model plus each **ONE** of the following CSF synaptic markers								
NPTX2	1.929 ± 0.770	.017	1.392 ± 0.443	.007	3.748 ± 1.318	.006	–0.854 ± 0.197	<.001
SNAP25	2.443 ± 1.075	.030	1.094 ± 0.602	.077	1.792 ± 1.761	.313	–0.483 ± 0.298	.111
Neurogranin	0.876 ± 0.692	.219	0.971 ± 0.357	.012	0.974 ± 1.345	.473	–0.210 ± 0.223	.353

NOTE. Results show slope terms for predictors of change in CVLT, MDRS, and CDR-sb over time. Results show the effects of adding each synaptic marker individually to the base model.

Abbreviations: CVLT, California Verbal Learning Test; MDRS, Dementia Rating Scale; CDR, Clinical Dementia Rating; NPTX2, Neuronal Pentraxin 2; SNAP25, Synaptosomal-associated protein 25.

### 3.2 ADNI replication cohort

Subject data are shown in Supplementary data (Supplementary Table 3, and predictors of progression in Supplementary Fig. 3 and Supplementary Table 4). We replicated the finding that NPTX2 added predictive value for the progression of RAVLT, ADAS-cog, and CDR-SB, in models that included demographic factors, Aβ1-42 and Tau. We similarly analyzed CSF Nfl as a predictor. The predictive power of NPTX2 for cognitive progression was at least comparable to that of Nfl and was consistent across measures of memory, global cognition, and CDR (Supplementary Table 4), whereas Nfl had strong predictive value for ADAS-cog and borderline significance for other tests. Findings were similar for both NPTX2 and Nfl after restricting the control, MCI, and AD subjects to those who were amyloid positive (CSF Aβ1-42 < 980 pg/mL). In survival analysis, NPTX2 was a stronger predictor of progression from MCI to AD than Nfl (Supplementary Fig. 4).

## 4 Discussion

This study extends previous efforts to link CSF biomarkers and cognition by focusing on markers of synaptic dysfunction across normal cognition, MCI, and mild AD-dementia. We assessed the diagnostic accuracy of the CSF biomarkers against the clinical diagnosis of normal cognition or AD (in the absence of amyloid PET or neuropathological data) using ROC analyses. Our findings of high accuracy of classification for CSF Aβ1-42 and Aβ1-42/Tau are consistent with many other studies [5]. We did not analyze Aβ1-40 because data are not available in both our study and ADNI, and we omitted P-Tau181 because levels correlate extremely highly with those of Tau (e.g., R > 0.9 in ADNI). We found that independent of Aβ1-42, levels of synaptic biomarkers expressed as ratios relative to Tau, in particular, NPTX2/Tau, achieved high classification accuracy. NPTX2 is decreased in the brain and CSF in AD, reflecting the loss of a key mechanism of synaptic homeostasis, whereas CSF Tau is increased, reflecting AD-related damage to neurons that may lead to regulated increases in the secretion of proteolytically cleaved Tau [34]. The ratio combining measures of these two processes seems to capture neurodegeneration in a way that correlates well with impaired cognitive performance, and can, therefore, track early stages of cognitive decline in AD.

Many studies have evaluated the temporal order in which cognitive tests change in biomarker-defined cohorts. Memory tests, such as the Free and Cued Selective Reminding Test (FCSRT) and CVLT-2, are among the most sensitive [26,35]. When we examined the relationship between different CSF markers of neurodegeneration and standardized measures of cognition, we found that ratios of candidate synaptic biomarkers to Tau correlated strongly with sensitive measures from the CVLT that probe memory acquisition and retention or the MDRS that assesses more general cognitive function. These correlations were much stronger than those for Tau alone and remained after AD amyloid biomarker classification was included. These correlational data, as well as the predictive data link amyloid and Tau, changes to synaptic changes as mediators of cognitive decline in AD. Although all three synaptic markers, NPTX2, SNAP25, and neurogranin, predicted progression on sensitive markers of cognition, NPTX2 added stronger and more consistent prognostic information in multivariate models.

A cluster of CSF biomarkers, namely Tau, neurogranin, SNAP25 in this study, and also alpha-synuclein, Visinin-like protein-1 (VILIP-1), GAP43 and Beta-secretase 1 (BACE-1) levels are increased in MCI and AD and correlate highly with one another [6,9,36,37]. Even in cognitively normal individuals, these markers clustered with structural brain measures (MRI) in a factor analysis [38]. The strong correlation between CSF markers of cytosolic (Tau), presynaptic (SNAP25) and dendritic (neurogranin) proteins suggests that they may represent a coordinated aspect of neurodegeneration. Correlations between CSF Aβ1-42 levels and all of the markers of neurodegeneration are relatively low, but in general, people classified as having low levels of Aβ1-42 show changes in CSF consistent with neurodegeneration, particularly at the MCI and mild AD stages [39].

Several biomarker discovery studies using mass spectrometry methods have identified changes in CSF levels of NPTX2 or NPTX1 in AD [12,40,41] and decreased Neuronal Pentraxin Receptor in Frontotemporal Dementia [42]. We measured CSF NPTX2 using a validated ELISA. CSF levels of NPTX2 had a low to medium correlation with CSF levels of Tau and other synaptic biomarkers and decreased as cognitive impairment increased. Combining NPTX2 and Tau as a ratio had high diagnostic accuracy for AD or MCI, and NPTX2 predicted cognitive decline in both mild AD and MCI. Even though it was measured by a different method in ADNI, CSF NPTX2 had similar prognostic value in that dataset (Supplementary Table 4). CSF NPTX2 is unique among synaptic biomarkers in that it assesses the function of excitatory synapses from pyramidal neurons that drive parvalbumin (PV) interneurons [13,14]. Neuronal pentraxins play a prominent role in synaptic homeostasis or organization [17,43]. In the mouse brain, Aβ increases excitability by reducing parvalbumin-positive interneuron function [44], and NPTX2 acts as a resilience mechanism by restoring circuit inhibition [19]. NPTX2-dependent excitatory synapses represent a small percentage of excitatory synapses in the brain, but they have a major impact on brain function, including control of rhythmicity important for episodic memory [17]. Reduced NPTX2 expression in the context of brain Aβ accumulation may play a role in increased activity in pyramidal neurons with resulting homeostatic diminution of excitatory synapses and consequent release of general synaptic markers. The observed relationship between CSF levels of NPTX2 and cognitive decline may indicate progressive changes in synaptic circuitry dependent on the extent of overall neurodegeneration. Whether or not these biomarker changes reflect the synaptic and neuronal loss or alteration in a unction that may be amenable to intervention or can serve as an outcome measure requires further study.

Regarding relationships to cognition, the ratios of CSF levels of synaptic markers to Tau showed robust correlations with both the MDRS and CVLT in MCI and AD in individuals with low CSF Aβ1-42 (Fig. 2 and Supplementary Table 2). Replicating and extending these findings in additional cohorts, including those in pre-MCI stages of AD, and examining changes in these CSF biomarkers in relationship to cognitive decline, will be future priorities. In addition, relationships among CSF levels of synaptic biomarkers, Tau, and neuroimaging markers of neurodegeneration should be studied across stages of AD. In ADNI, CSF NPTX2 was reported to predict 12-month medial temporal lobe atrophy rates on MRI [41]. We extended these analyses, using Elecsys data for Tau and Aβ1-42, and found that in MCI and AD subjects with low CSF Aβ1-42, NPTX2 was a significant predictor of atrophy rates, whereas Nfl was not (data not shown).

Overall, these results support the use of NPTX2 and other CSF biomarkers as aids to refine prognosis in MCI and AD, with potential applications in clinical or research studies. A limitation of our study is that the UCSD and ADNI cohorts may not generalize to the community; therefore, replication and extension of these findings is important.

In AD, neuropathology builds up for years before the development of MCI. Prior CSF biomarker studies suggest that levels of neurogranin, a ratio of NPTX2 to another synaptic peptide, ratios between other synaptic peptides (e.g., neurogranin/BACE1), or CSF levels of a panel of synaptic proteins may correlate with cognitive changes before the advent of clinically recognizable MCI [11,16,45]. Our results support this emerging concept by showing relationships between synaptic biomarkers in CSF and cognition in cognitively normal elders, but further research is clearly warranted. As more molecular biomarkers related to synaptic integrity, neuroinflammation, vascular injury, and other processes are identified in CSF [46], these will support a more detailed map of neurodegeneration. This will enable more precise staging and prediction of change during the decades in which AD runs its course from preclinical to symptomatic stages.

**Research in context**

1. Systematic review: Synaptic damage is prominent in Alzheimer's Disease (AD), including in its early stages. We searched PubMed for publications on synaptic biomarkers measured in CSF in relation to cognition and progression in Mild Cognitive Impairment and Alzheimer's Disease.
2. Interpretation: We measured the novel synaptic marker NPTX2 in CSF with an ELISA assay and included additional data from ADNI that measured a NPTX2-related peptide in CSF. We found that CSF NPTX2 was decreased in MCI and AD and weakly correlated with CSF levels of Tau, unlike other synaptic markers (neurogranin and SNAP25), which were increased in CSF and strongly correlated with Tau. CSF NPTX2 improved correlations between CSF biomarkers and measures of cognition. NPTX2 substantially increased the ability of baseline CSF biomarkers to predict progression in MCI and AD. NPTX2 is a novel synaptic biomarker that may improve prognostic ability in AD.
3. Future directions: Extend and replicate these findings in larger cohorts, including preclinical AD. Examine NPTX2 in other neurodegenerative disorders, for example, Frontotemporal Lobar Degeneration and Dementia with Lewy Bodies.
