# Supplementary material for: Synaptic biomarkers in CSF aid in diagnosis, correlate with cognition and predict progression in MCI and Alzheimer's disease
Source: Alzheimers Dement (N Y). 2019 Dec 9;5:871–82. doi: 10.1016/j.trci.2019.11.002 (PMC6911971; doi:10.1016/j.trci.2019.11.002)
Supplement: Supplementary Data [file mmc1.docx]

**SUPPLEMENTARY MATERIAL**

**Supplementary Methods: SNAP25 assay**

All Simoa measurements were performed on a fully automated Simoa HD-1 Analyzer (Quanterix). The microparticles, coated with AD404DEV capture antibody, specific for the N-terminus of SNAP25 were diluted in Bead Diluent (Quanterix) 1:100.The number of active beads was reduced and replaced by non-reactive microparticles (Helper Beads, Quanterix). Approximately 50% non-functional and 50% functional microparticles per sample were used. The biotinylated ADX405detection antibody, its epitope determined by SNAP25 sequences, L22-L33, was diluted in Detector Diluent (Quanterix) to a working concentration of 1 μg/mL. Streptavidin-β-galactosidase concentrate was diluted in SBG Diluent (Quanterix) to a working concentration of 50 pM. Resorufin β-D-galactopyranoside substrate was used as provided by Quanterix. The assay configuration protocol was a two-step assay. In the first step, 25 μL of the microparticles solution, 20 μL of detector antibody, and a 152 μL sample were incubated for 60 min (80 cadences) in a reaction cuvette (Quanterix), followed by several wash steps. In the second step, 100 μL of SβG was added and incubated for 5min and 15 s (7 cadences), followed by several wash steps. 672 µL microliters of RGP substrate solution were added to the microparticles, mixed, and loaded onto the Simoa disc array. The array was then sealed with oil and microparticles were imaged. Automated analysis was done by the HD-1 Analyzer software version 1.5 (Quanterix). The calibration curve covered a concentration range of 2,5-100 pg/ml.

**Supplementary Figure 1: ROC curves for individual CSF biomarkers and for ratios, classifying AD and normal cognition groups in the UCSD cohort**

**
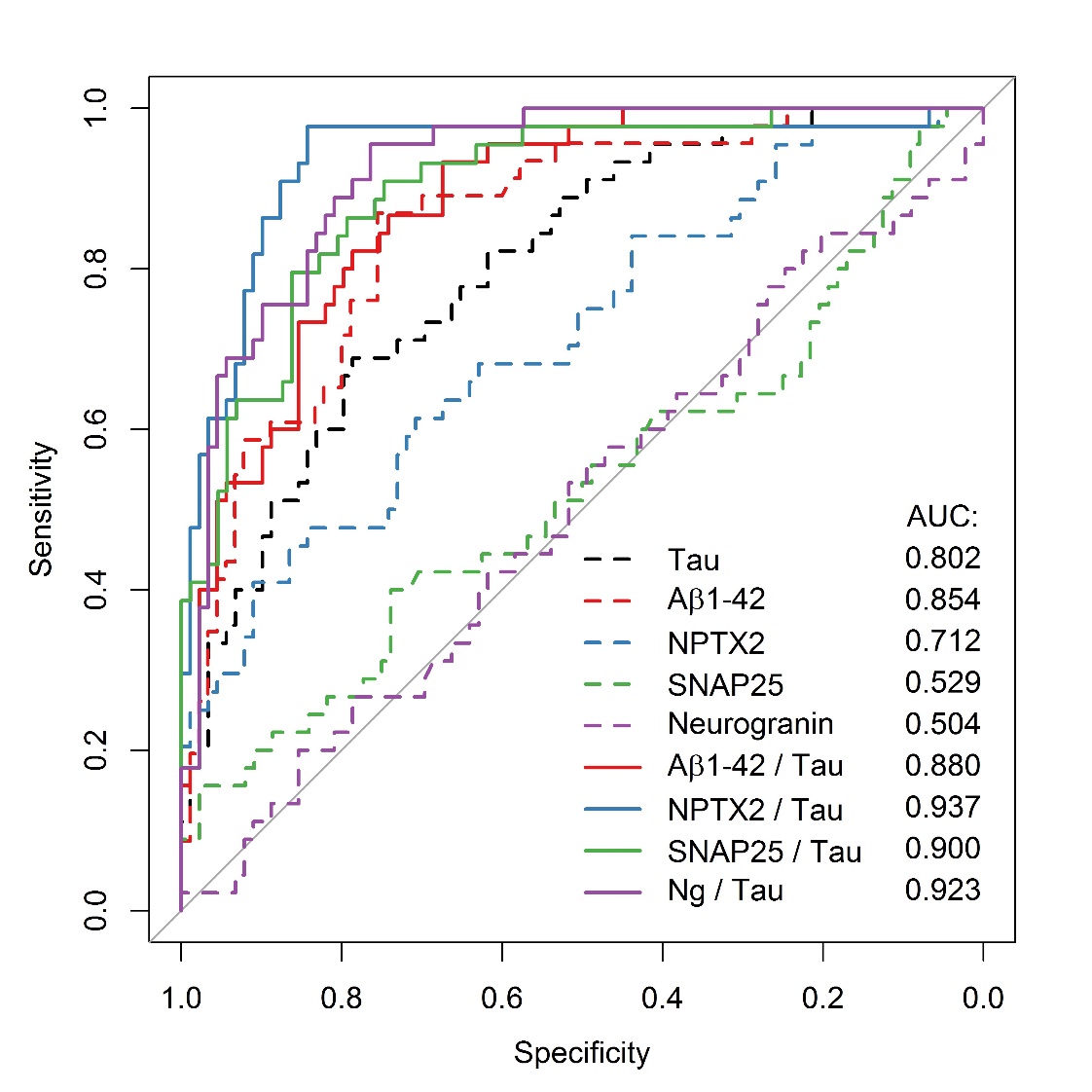
**

Receiver Operating Characteristic **(**ROC) curves for classification of NC vs AD for individual biomarkers and selected ratios. Dashed lines are used for individual markers, solid lines of the same color for their ratios.

**Supplementary Figure 2: ROC curves for individual CSF biomarkers and for ratios, classifying normal cognition (NC), stable and progressing MCI groups in the UCSD cohort**


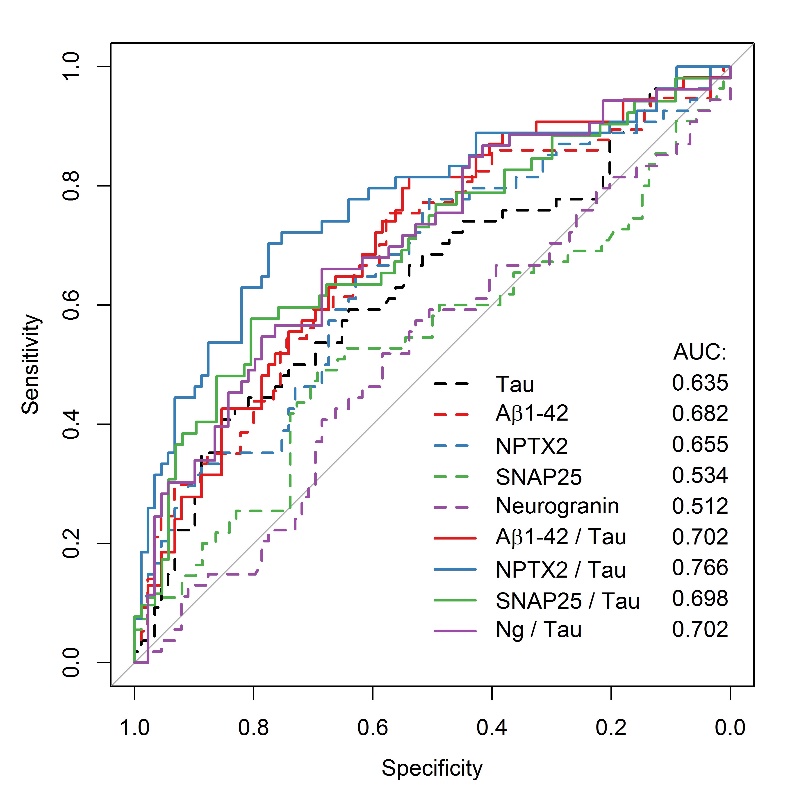

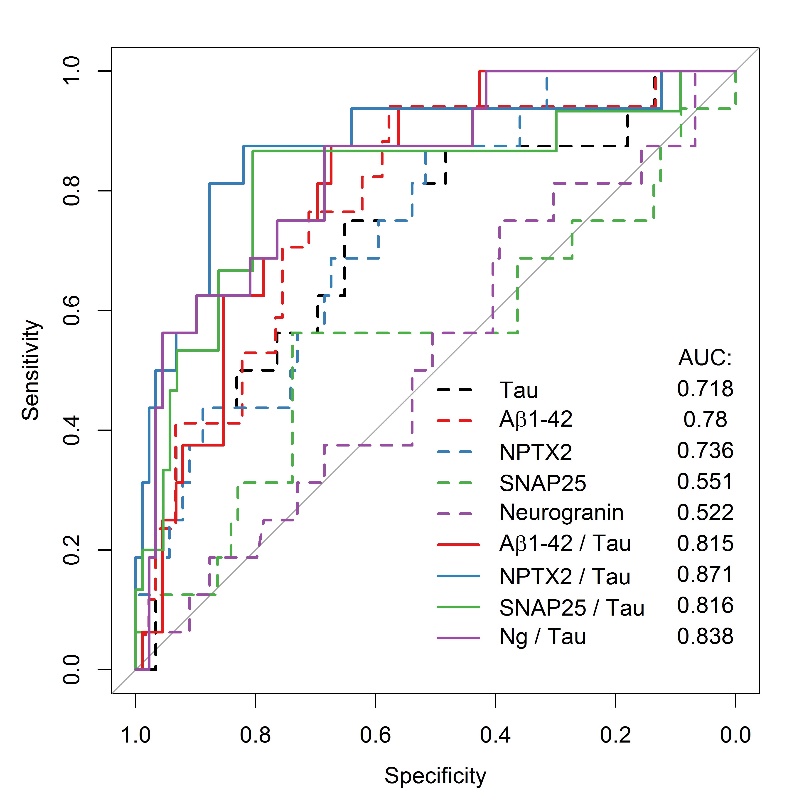

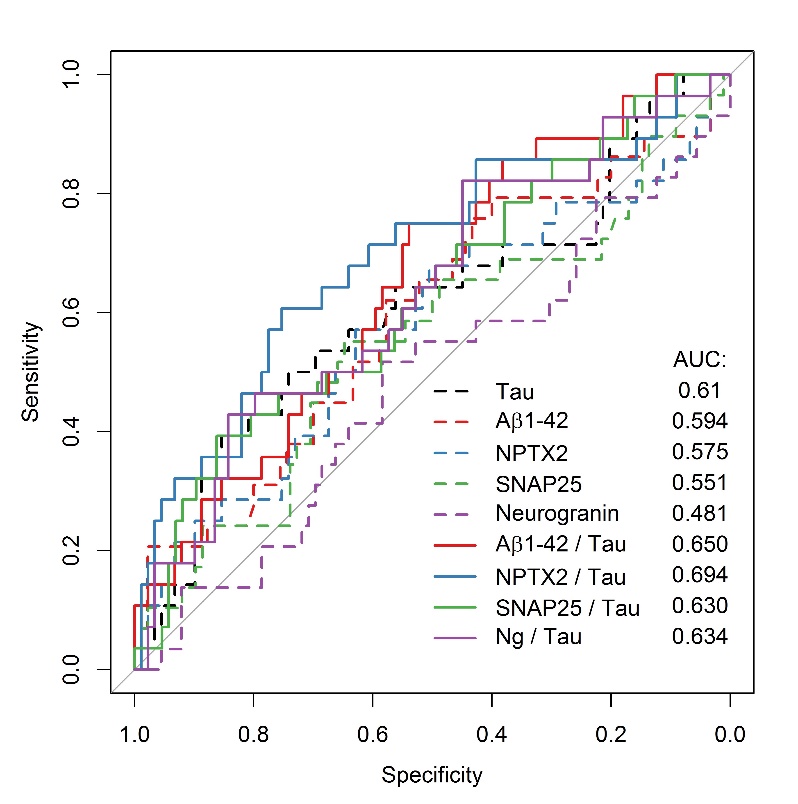


NC versus all MCI

NC versus progressing MCI

NC versus stable MCI

**Supplementary Table 1: Diagnostic discrimination between AD and controls for biomarkers**

**and their combinations, UCSD cohort**

|  | AUC [95% CI] | Optimal threshold | Specificity  [95% CI] | Sensitivity [95% CI] |
| --- | --- | --- | --- | --- |
| Aβ1-42 | 0.854  [0.789 - 0.92] | 480.536 | 0.756  [0.478 – 0.833] | 0.870  [0.564 – 0.956] |
| Tau | 0.802  [0.726 - 0.878] | 502.177 | 0.787  [0.561 – 0.876] | 0.689  [0.467 – 0.822] |
| Aβ1-42/Tau | 0.880  [0.824 - 0.936] | 0.917 | 0.787  [0.809 – 0.955] | 0.822  [0.864 – 1.000] |
| NPTX2 | 0.712  [0.617 - 0.807] | 731.325 | 0.708  [0.438 – 0.832] | 0.614  [0.386 – 0.750] |
| SNAP25 | 0.529  [0.419 - 0.639] | 36.144 | 0.739  [0.433 – 0.841] | 0.400  [0.156 – 0.533] |
| Neurogranin | 0.504  [0.398 - 0.609] | 167.784 | 0.854  [0.640 – 0.932] | 0.200  [0.022 – 0.311] |
| NPTX2/ Aβ1-42 | 0.570  [0.456 - 0.684] | 1.645 | 0.652  [0.236 – 0.775] | 0.591  [0.364 – 0.705] |
| SNAP25/ Aβ1-42 | 0.822  [0.751 - 0.893] | 0.057 | 0.682  [0.432 – 0.784] | 0.867  [0.644 – 0.956] |
| Neurogranin/ Aβ1-42 | 0.748  [0.660 - 0.835] | 0.557 | 0.685  [0.427 – 0.786] | 0.756  [0.533 – 0.867] |
| NPTX2/Tau | 0.937  [0.888 - 0.986] | 1.871 | 0.843  [0.337 – 0.921] | 0.977  [0.750 – 1.000] |
| SNAP25/Tau | 0.900  [0.847 - 0.954] | 0.064 | 0.862  [0.713 – 0.931] | 0.795  [0.523 – 0.932] |
| Neurogranin/Tau | 0.923  [0.879 - 0.966] | 0.702 | 0.764  [0.505 – 0.843] | 0.956  [0.764 – 1.000] |

**Supplementary Table 2. Cross-sectional predictors of cognitive measures, UCSD cohort**

**2a: Predictors of CVLT Immediate Recall in subjects classified by CSF Aβ1-42/Tau**

| CVLT | All Participants | | Aβ1-42/Tau Negative | | Aβ1-42/Tau Positive | |
| --- | --- | --- | --- | --- | --- | --- |
|  | *Beta ± Std. Error* | *p value* | *Beta ± Std. Error* | *p value* | *Beta ± Std. Error* | *p value* |
| Age | 0.100 ± 0.363 | 0.473 | -0.456 ± 0.392 | 0.020 | 0.249 ± 0.649 | 0.225 |
| Sex | 7.900 ± 0.363 | < 0.001 | 13.068 ± 0.392 | <0.001 | 0.041 ± 0.649 | 0.990 |
| Education | 0.327 ± 0.363 | 0.369 | 0.266 ± 0.392 | 0.499 | -0.056 ± 0.649 | 0.932 |
| APOE e4 | 0.498 ± 0.363 | 0.805 | 0.177 ± 0.392 | 0.938 | 0.838 ± 0.649 | 0.817 |
| AB42 | 0.012 ± 0.363 | 0.001 | 0.003 ± 0.392 | 0.516 | 0.035 ± 0.649 | 0.025 |
| Tau | -0.017 ± 0.363 | < 0.001 | -0.010 ± 0.392 | 0.221 | -0.012 ± 0.649 | 0.030 |
| Above model plus **ONE** of the following: | | | | | | |
| NPTX2 | 0.011 ± 0.002 | < 0.001 | 0.007 ± 0.003 | 0.007 | 0.015 ± 0.003 | < 0.001 |
| SNAP25 | 0.287 ± 0.147 | 0.052 | 0.002 ± 0.182 | 0.991 | 0.315 ± 0.239 | 0.193 |
| Neurogranin | 0.040 ± 0.008 | < 0.001 | 0.048 ± 0.013 | <0.001 | 0.031 ± 0.011 | 0.010 |

**2b: Predictors of CVLT Delayed Recall in subjects classified by CSF Aβ1-42/Tau**

| CVLT delay | All Participants | | Aβ1-42/Tau Negative | | Aβ1-42/Tau Positive | |
| --- | --- | --- | --- | --- | --- | --- |
|  | *Beta ± Std. Error* | *p value* | *Beta ± Std. Error* | *p value* | *Beta ± Std. Error* | *p value* |
| Age | 0.045 ± 0.235 | 0.614 | -0.382 ± 0.274 | 0.006 | 0.234 ± 0.394 | 0.062 |
| Sex | 4.269 ± 0.235 | < 0.001 | 6.744 ± 0.274 | <0.001 | 0.825 ± 0.394 | 0.689 |
| Education | 0.251 ± 0.235 | 0.287 | 0.277 ± 0.274 | 0.314 | -0.01 ± 0.394 | 0.980 |
| APOE 4 | -0.469 ± 0.235 | 0.719 | -0.282 ± 0.274 | 0.858 | -0.487 ± 0.394 | 0.824 |
| Aβ1-42 | 0.008 ± 0.235 | 0.001 | 0.002 ± 0.274 | 0.499 | 0.016 ± 0.394 | 0.094 |
| Tau | -0.010 ± 0.235 | < 0.001 | -0.008 ± 0.274 | 0.166 | -0.005 ± 0.394 | 0.117 |
| Above model plus **ONE** of the following: | | | | | | |
| NPTX2 | 0.005 ± 0.001 | 0.001 | 0.002 ± 0.002 | 0.21 | 0.007 ± 0.002 | 0.001 |
| SNAP25 | 0.271 ± 0.093 | 0.004 | 0.018 ± 0.127 | 0.888 | 0.361 ± 0.138 | 0.012 |
| Neurogranin | 0.022 ± 0.005 | < 0.001 | 0.034 ± 0.009 | <0.001 | 0.015 ± 0.007 | 0.035 |

**2c: Predictors of MDRSin subjects classified by CSF Aβ1-42/Tau**

| MDRS | All Participants | | Aβ1-42/Tau Negative | | Aβ1-42/Tau Positive | |
| --- | --- | --- | --- | --- | --- | --- |
|  | *Beta ± Std. Error* | *p value* | *Beta ± Std. Error* | *p value* | *Beta ± Std. Error* | *p value* |
| Age | 0.088 ± 0.279 | 0.469 | 0.004 ± 0.320 | 0.980 | 0.118 ± 0.480 | 0.534 |
| Sex (Female) | 4.552 ± 0.279 | 0.006 | 3.470 ± 0.320 | 0.047 | 7.107 ± 0.480 | 0.028 |
| Education | 0.915 ± 0.279 | 0.001 | 0.181 ± 0.320 | 0.572 | 1.670 ± 0.480 | 0.001 |
| APOE e4 | 2.065 ± 0.279 | 0.246 | 3.581 ± 0.320 | 0.068 | -0.922 ± 0.480 | 0.787 |
| Aβ1-42 | 0.012 ± 0.279 | < 0.001 | 0.009 ± 0.320 | 0.013 | 0.018 ± 0.480 | 0.229 |
| Tau | -0.011 ± 0.279 | < 0.001 | -0.009 ± 0.320 | 0.190 | -0.010 ± 0.480 | 0.001 |
| Above model plus **ONE** of the following: | | | | | | |
| NPTX2 | 0.007 ± 0.002 | < 0.001 | 0.008 ± 0.002 | 0.001 | 0.009 ± 0.003 | 0.008 |
| SNAP25 | 0.159 ± 0.100 | 0.115 | 0.129 ± 0.154 | 0.405 | 0.208 ± 0.161 | 0.201 |
| Neurogranin | 0.021 ± 0.007 | 0.002 | 0.034 ± 0.012 | 0.004 | 0.022 ± 0.010 | 0.029 |

**Supplementary Table 3. Demographic, cognitive and biomarker data from the ADNI cohort**

|  | AD (n = 66) | MCI (n = 140) | NC (n = 86) | p value |
| --- | --- | --- | --- | --- |
| Age (years) | 75.1 ± 7.6 | 74.7 ± 7.2 | 75.7 ± 5.5 | 0.563 |
| Female (Number (%)) | 28 (42%) | 44 (31%) | 43 (50%) | 0.019^a^ |
| Education (years) | 15 ± 3 | 16.1 ± 2.9 | 15.7 ± 3 | 0.058 |
| MMSE (0-30) | 23.5 ± 1.9 | 26.9 ± 1.7 | 29 ± 1 | <0.001^a,b,c^ |
| ADAS-cog (errors) | 18.7 ± 6.6 | 11.7 ± 4.3 | 6.1 ± 2.9 | <0.001^a,b,c^ |
| AVLT trials 1-5 (0-80) | 22.9 ± 7.5 | 30 ± 8.6 | 43.3 ± 8.4 | <0.001^a,b,c^ |
| AVLT Delayed Recall  (0-12) | 0.8 ± 1.9 | 2.4 ± 3 | 7.4 ± 3.5 | <0.001^a,b,c^ |
| CDR Sum of Boxes  (0-18) | 4.3 ± 1.6 | 1.5 ± 0.9 | 0 ± 0.1 | <0.001^a,b,c^ |
| APOE e4 % positive | 47 (71%) | 74 (53%) | 20 (23%) | <0.001^a,b,c^ |
| Aβ1-42 (pg/mL) | 628.1 ± 344.6 | 814.9 ± 449 | 1303 ± 658.9 | <0.001^a,b,c^ |
| Tau (pg/mL) | 359.6 ± 129.2 | 317.4 ± 118.1 | 240.9 ± 76.3 | <0.001^a,b,c^ |
| Aβ1-42 /Tau | 2 ± 1.4 | 3.1 ± 2.3 | 5.8 ± 2.6 | <0.001^a,b,c^ |
| NPTX2 (pg/mL) | 10.3 ± 0.9 | 10.6 ± 0.7 | 10.7 ± 0.5 | 0.003^b,c^ |
| Nfl (pg/mL) | 1834.7 ± 1132.1 | 1568.9 ± 1267.4 | 1103.4 ± 376.5 | <0.001^a,b^ |

AD = Alzheimer’s Disease, MCI = Mild Cognitive Impairment, NC = normal cognition.

MMSE = Mini-Mental State Examination; ADAS-cog = Alzheimer’s Disease Assessment Scale – Cognitive Subscale 11; RAVLT = Rey Auditory Verbal Learning Test; CDR = Clinical Dementia Rating; NPTX2 = Neuronal Pentraxin 2; Nfl = Neurofilament Light Chain. NPTX2 levels are log-transformed by the ADNI Biomarker Core.

Results are presented as mean ± standard deviation.

^a^ post-hoc difference (p < 0.05) between NC and MCI
^b^ post-hoc difference (p < 0.05) between NC and AD
^c^ post-hoc difference (p < 0.05) between MCI and AD

**Supplementary Table4: ADNI longitudinal model results**

**4a. Longitudinal model results for all subjects with MCI and AD**

|  | RAVLT Immediate | | RAVLT Delay | | ADAS-cog | | CDR-sb | |
| --- | --- | --- | --- | --- | --- | --- | --- | --- |
|  | *Beta ± Std. Error* | *p value* | *Beta ± Std. Error* | *p value* | *Beta ± Std. Error* | *p value* | *Beta ± Std. Error* | *p value* |
| Age | 0.026 ± 0.025 | 0.295 | -0.010 ± 0.007 | 0.182 | -0.036 ± 0.031 | 0.241 | 0 ± 0.011 | 0.984 |
| Sex | 0.515 ± 0.385 | 0.184 | 0.204 ± 0.113 | 0.071 | -1.025 ± 0.478 | 0.034 | -0.117 ± 0.177 | 0.509 |
| Education | -0.109 ± 0.056 | 0.056 | 0.007 ± 0.016 | 0.672 | 0.002 ± 0.072 | 0.974 | 0.039 ± 0.027 | 0.151 |
| APOE e4 | 0.030 ± 0.399 | 0.940 | 0.136 ± 0.117 | 0.248 | -0.088 ± 0.498 | 0.860 | -0.243 ± 0.183 | 0.187 |
| Aβ1-42 | 1.034 ± 0.244 | < 0.001 | 0.321 ± 0.072 | < 0.001 | -1.078 ± 0.320 | 0.001 | -0.371 ± 0.117 | 0.002 |
| Tau | -0.263 ± 0.172 | 0.129 | -0.057 ± 0.050 | 0.255 | 0.279 ± 0.213 | 0.191 | 0.113 ± 0.078 | 0.147 |
| Plus **ONE** of the following: | | | | | | | | |
| NPTX2 | 1.059 ± 0.200 | < 0.001 | 0.161 ± 0.061 | 0.009 | -1.546 ± 0.229 | < 0.001 | -0.264 ± 0.095 | 0.006 |
| Nfl | -0.244 ± 0.143 | 0.090 | -0.068 ± 0.041 | 0.104 | 0.534 ± 0.196 | 0.008 | 0.153 ± 0.073 | 0.039 |

**4b.Longitudinal model results for amyloid positive subjects with MCI and AD**

|  | RAVLT Immediate | | RAVLT Delay | | ADAS-cog | | CDR-sb | |
| --- | --- | --- | --- | --- | --- | --- | --- | --- |
|  | *Beta ± Std. Error* | *p value* | *Beta ± Std. Error* | *p value* | *Beta ± Std. Error* | *p value* | *Beta ± Std. Error* | *p value* |
| Age | 0.047 ± 0.030 | 0.129 | -0.001 ± 0.008 | 0.876 | -0.045 ± 0.039 | 0.252 | -0.002 ± 0.014 | 0.895 |
| Sex | 0.344 ± 0.449 | 0.447 | 0.296 ± 0.121 | 0.015 | -1.065 ± 0.585 | 0.071 | -0.140 ± 0.213 | 0.515 |
| Education | -0.156 ± 0.066 | 0.022 | -0.003 ± 0.018 | 0.867 | 0.013 ± 0.09 | 0.885 | 0.039 ± 0.033 | 0.241 |
| APOE e4 | 0.022 ± 0.437 | 0.960 | 0.064 ± 0.117 | 0.588 | 0.335 ± 0.569 | 0.558 | -0.237 ± 0.208 | 0.259 |
| Tau | -0.246 ± 0.203 | 0.230 | -0.030 ± 0.054 | 0.581 | 0.242 ± 0.266 | 0.365 | 0.102 ± 0.096 | 0.292 |
| Plus **ONE** of the following: | | | | | | | | |
| NPTX2 | 1.194 ± 0.228 | < 0.001 | 0.134 ± 0.066 | 0.047 | -1.686 ± 0.278 | < 0.001 | -0.290 ± 0.115 | 0.013 |
| Nfl | -0.465 ± 0.221 | 0.037 | -0.072 ± 0.059 | 0.222 | 0.674 ± 0.284 | 0.019 | 0.226 ± 0.102 | 0.029 |

Results show slope terms for predictors of change in CVLT, MDRS and CDR-sb over time. Results show effects of adding each synaptic marker individually to the base model.

**Supplementary Figure 3. CSF NPTX2 and NFL and clinical progression of ADNI MCI and AD subjects**
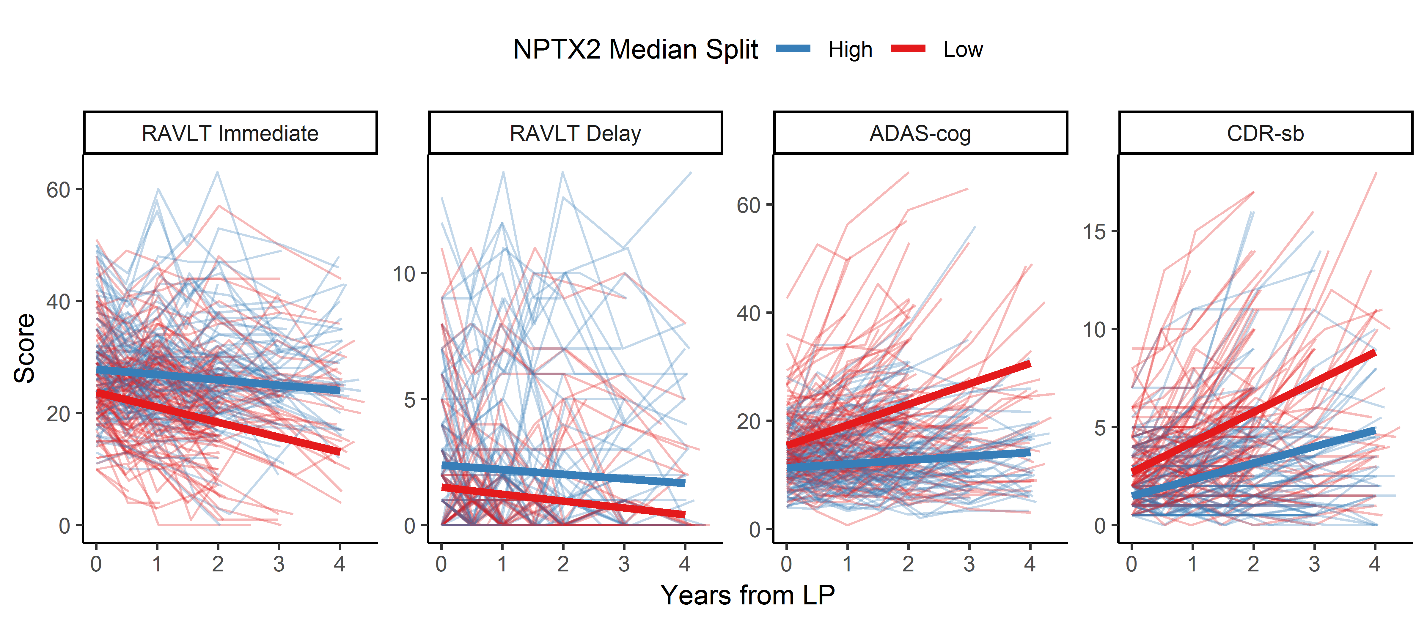


**
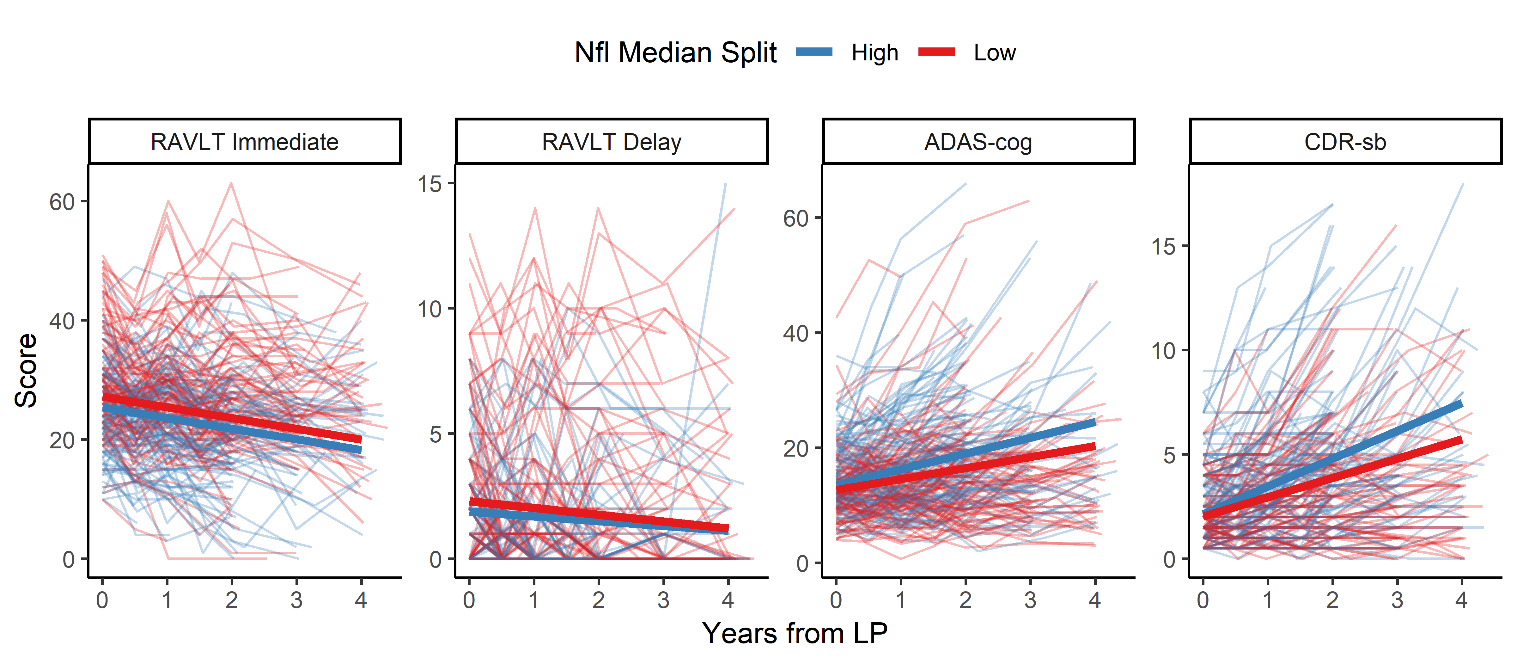
**

**Supplementary Figure 4: Predictors of conversion from MCI to dementia in ADNI subjects**

**
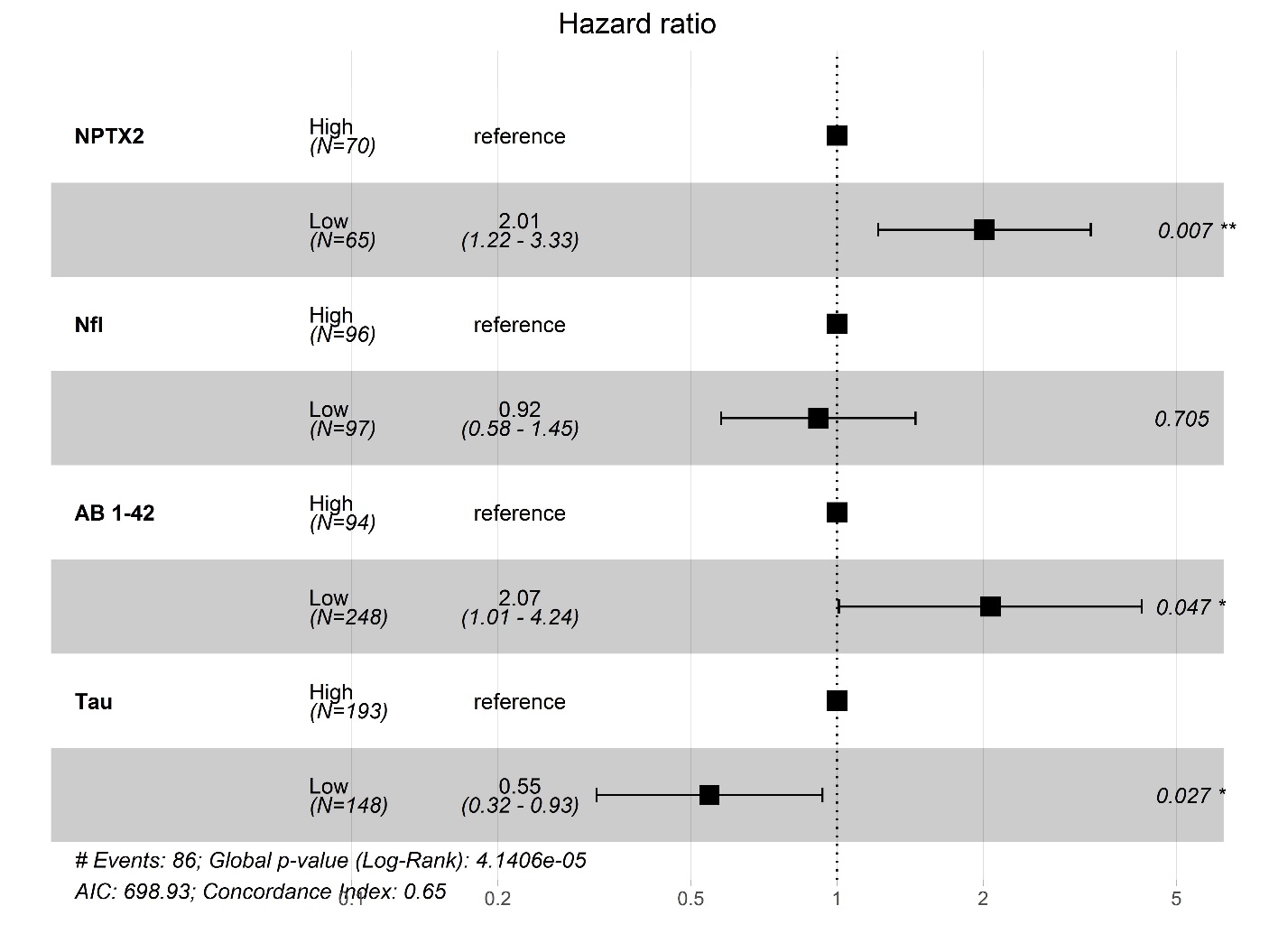
**

Cox proportional hazard model, ADNI MCI subjects, followed for up to 5 years, with progression to dementia as outcome. Predictors were age, sex, and CSF biomarkers. With Aβ1-42 as a reference, lower Tau was protective and higher NPTX2 increased thehazard.
